# Mechanism of SQQX Decoction's Protective Effect on SHR: A Serum Metabolomics-Based Analysis

**DOI:** 10.1155/2020/8856943

**Published:** 2020-12-09

**Authors:** Jiayun Wu, Lingling Li, Lin Li, Yanjun Li, Xiang Xiao, Jiajun Qiao, Rui Sun, Deshuang Yang, Ruiqi Yao, Li Huang

**Affiliations:** ^1^Beijing University of Chinese Medicine, Beijing 100029, China; ^2^Department of Integrative Cardiology, China-Japan Friendship Hospital, Beijing 100029, China; ^3^Rizhao International Heart Hospital, Shandong 276826, China

## Abstract

SangQiQingXuan (SQQX) decoction is a pharmaceutical preparation exerting good therapeutic efficacy on high blood pressure (BP) and has widely been accepted in primarily hypertensive patients as a herbal formula prescribed by Professor Li Huang from China-Japan Friendship Hospital according to her 30-year clinical experience. A previous study showed that SQQX could reduce BP by decreasing levels of many inflammatory factors such as transforming growth factor beta (TGF*β*) and elevating peroxisome proliferator activated receptor (PPAR) expression. However, a research focusing on SQQX's protection against HTN from a metabolomic perspective has never been done before. This study aimed to figure out the metabolic profiling variations due to oral administration of SQQX in spontaneous hypertensive rat (SHR) models and to find out the optimal dosage of SQQX. SHR in the intervention group orally received SQQX extract of three doses, namely, the low- (5.25 g/kg/d), middle- (10.5 g/kg/d), and high-dosage groups (21 g/kg/d) for 90 days. Rats were sacrificed at the end of the experiment, and their serum was collected for further examination. Serum metabolic profiling variations were analyzed using ultraperformance liquid chromatography coupled with tandem mass spectrometry (UPLC/MS). Results showed that dealing with SQQX remarkably decreased systolic blood pressure (SBP) of SHRs and the high-dosage group was with the best therapeutic effect where a total of 11 metabolites were markedly changed in contrast to the model group. Orthogonal partial least square discriminant analysis (OPLS-DA) score plot showed that the 5 groups of serum samples were divided into 5 categories, and the metabolic trajectory of the high-dosage SQQX group was inclined to move to the control group. Glycochenodeoxycholic acid, nicotinamide-N-oxide, and tryptophan betaine might be biomarkers that specifically marked the protective effects of SQQX against high BP mainly involving in cholesterol metabolism, primary bile acid biosynthesis, bile secretion, and nicotinate and nicotinamide metabolism. To conclude, SQQX has a protective effect on SHR, which may be partially correlated to restoration of perturbed metabolism in serum.

## 1. Introduction

Hypertension (HTN) is the most common cardiovascular disease in clinical practice and is one of the most important risk factors that plays a crucial role in cardiovascular and cerebrovascular diseases such as stroke, coronary heart disease, and diabetes [1]. It is reported that 30% of the world's population suffers from HTN, and this number is expected to rise to about 50% by 2025 [2]. And according to the 2018 Report on Cardiovascular Diseases in China, the number of hypertensive patients in China is 245 million out of 290 million patients diagnosed with cardiovascular diseases, taking up to 85% [3]. Moreover, the target organ damage caused by HTN has already become a leading cause of patient deaths [4, 5]. The death number caused by it is over 7 million per year, and it is still at a rise. The high incidence and mortality make treatment of HTN of real urgency. Current treatments for HTN are primarily calcium antagonist, angiotensin converting enzyme inhibitors, angiotensin II receptor blockers, beta blockers, and diuretics. Despite effective results in clinical cases and animal models, their side effects and drug-resistant problems remained to be solved. Besides, as a result of both genetic susceptibility and environmental factors, there is no perfect and unified understanding on pathogenic mechanisms of HTN. Traditional Chinese medicine (TCM), as an integrative medicine, evidence has showed its great effect on HTN as it could not only reduce BP but also improve the overall condition of patients with less side effects or drug resistance by supplementing deficiency and removing excess in their bodies to rebalance yin and yang.

SQQX decoction is a hospital preparation made by Professor Huang Li of China-Japan Friendship Hospital based on her 3-decade clinical experience, and its curative effect has been well received by hypertensive patients. Composed of Herba Taxilli (Sang-Jisheng), Fructus Lycii (Gou-Qi), Cortex Eucommiae (Du-Zhong), Semen Cassiae (Jue-Mingzi), Radix et Rhizoma Salviae Miltiorrhizae (Dan-Shen), Rhizoma Alismatis (Ze-Xie), Radix Puerariae Lobatae (Ge-Gen), and Flos Chrysanthemiindici (Ye-Juhua), SQQX has the ability to resolve phlegm, activate the blood circulation, and tonify the kidney and spleen to attenuate hypertensive target organ damage as in TCM theory: HTN is a result of deficiency in the liver and kidney yin and dysfunction of the spleen, along with phlegm turbidity and blood stasis. A previous study has proved that SQQX could decrease BP and prevent ventricular remodeling, exerting vasoprotective, cardioprotective, and renoprotective effects on SHR [6–8]. And these effects are closely related to the up-stream inflammatory factors of TGF*β* and PPARs signaling ways [7]. However, the effect of SQQX on serum metabolites of SHR by UPLC/MS has never accomplished before.

Metabolomics has been a powerful way to screen out the significant biochemical metabolites in relation with the pathogenesis of diseases and could help make clinical diagnosis and track patients following up or even effects of therapeutic methods by detecting levels of these special small molecules [9]. Metabolome refers to a collection of all metabolites in an organism's cell, tissue, or organ, which partly shares the same holistic view with TCM and gradually becomes an important tool to investigate the substance alteration and action mechanism of TCM [10]. In recent years, many research studies focused on coping HTN with TCM herbs or formulas have been conducted using ultraperformance liquid chromatography coupled with tandem mass spectrometry (UPLC/MS) providing a new way to reveal effects of TCM [11–14].

In this study, SHR was used as a model to simulate human HTN disorders to the greatest extent. General situation and BP were observed to evaluate stability of models and effects of SQQX. At last, serum metabolic profiling change after SQQX intervention was determined using broad-spectrum targeted metabolomic approach, aiming to explain the potential mechanisms of SQQX from a perspective of small molecular biological metabolites for the first time.

## 2. Materials and Methods

### 2.1. Chemicals and Reagents

Methanol and acetonitrile were purchased from Merck (Merck, USA); standard substance was bought from Sigma-Aldrich (St.Louis, MO, USA). SQQX is composed of Herba Taxilli, Fructus Lycii, Cortex Eucommiae, Semen Cassiae, Radix et Rhizoma Salviae Miltiorrhizae, Rhizoma Alismatis, Radix Puerariae Lobatae, and Flos Chrysanthemiindici (Supplementary Table S1). All the botanical names and their correspondent life science identifier (LSID) on the International Plant Names Index (IPNI) have been recorded and can be validated by searching http://mpns.kew.org. All herbal materials were purchased from Beijing Tong Ren Tang (Beijing, China) and prepared by the Pharmaceutical Department of China-Japan Friendship Hospital as previously described [7], except for that the granule making and drying processes were removed. Liquid chromatography-mass spectrometry (LC-MS) analysis was conducted to confirm main gradients of SQQX extract, as indicated in Supplementary Figure S1.

### 2.2. Animals

All animal experiments were carried out in accordance with protocols approved by the Ethics Committee of China-Japan Friendship Hospital (no.180209), and all efforts were made to minimize animal suffering. All animals were purchased from Beijing Vital River Laboratory Animal Technology Company (certificate no. SCXK 2016-0043) and were fed with standard feed. Animal living temperature was controlled at 23°C∼28°C, humidity was at 50 ± 5%, and the cycle of light and darkness was 12 h/12 h. There were 3 rats from the same group in each cage, and food and drinking water could be freely obtained. Eight-week-old male SHRs (180 g∼200 g) were randomly separated into the following 4 groups: model group (*n* = 6), low-dosage SQQX group (*n* = 6), middle-dosage SQQX group (*n* = 6), and high-dosage SQQX group (*n* = 6). Meanwhile, six WKY rats aged 8 weeks (185 g∼205 g) were served as the control group (*n* = 6). After 4-week BP monitoring, rats in the control group (245∼250 g) and the model group (235∼246 g) began to be fed with clean water while SQQX groups (235∼246 g) were administered with 5.25 g/kg/d, 10.5 g/kg/d, and 21 g/kg/d SQQX extract by gastric irrigation for 90 days since the BP of SHRs was relatively stable. All rats were anesthetized (sodium pentobarbital, 75 mg/kg, intraperitoneal injection), and blood samples were obtained from abdominal aorta. Rats were euthanized by cervical dislocation without regaining consciousness.

### 2.3. General Condition of WKY and SHR

Body weight of rats was measured, and changes in mental state, hair color, and fights, along with stool character of rats were observed and recorded before and after SQQX intervention.

### 2.4. BP Analysis

The caudal artery BP of rats, especially SBP, was measured once a week at the same time period and in the same platform channel in a quiet room (so that the rats could fully adapt to the platform stimulation to ensure the stability and reliability of the SBP value). The SBPs before and after 90-day oral administration were used for analysis. After staying in the box on the platform for 10 to 15 mins, tail SBP of rats was detected using BP 2000 (Visitech Systems, USA). All rats at each time point were measured 10 times, and the mean values of 6 measurements whose waveforms were more in line with the criteria were taken as the final BP values.

### 2.5. Serum Extraction for UPLC-MS Analysis

Serum samples were taken out from the refrigerator at −80°C, thawed on ice, and after thawing, they were vortexed for 10 s to get mixed. Then, 3 volumes of ice-cold methanol were added to 1 volume of plasma/serum. Specifically, 50 *μ*L of the sample was taken in an EP tube and 150 *μ*L of precooled ice methanol was added (containing 1 *μ*g/mL of 2-chlorophenylalanine as an internal standard). The mixture was thoroughly vortexed for 3 min and then centrifugated at a speed of 12000 rpm at 4°C for 10 min. After centrifugation, the supernatant was put into another new EP tube and centrifugated at 12000 rpm, 4°C for 5 min. Finally, the supernatant was collected again and used for further UPLC/MS analysis.

### 2.6. Sample Extraction of Quality Control Sample

To guarantee the quality and validity of the bioanalytical data obtained from a broad-spectrum targeted metabolomic method, quality control (QC) samples were prepared from a mixture of pooled serum sample extracts to monitor the repeatability of analytical samples under the same processing method. In the process of instrument analysis, QC specimens were inserted into every 10 experimental samples throughout the whole analysis procedure. By performing overlapping display analysis on the total ion chromatography (TIC) diagram of different QC samples using mass spectrometry, the repeatability of the extraction and detection of metabolites could be judged.

### 2.7. UPLC-MS Analysis

The sample extracts were analyzed using an LC-ESI-MS/MS system (UPLC, Shim-pack UFLC SHIMADZU CBM A system, https://www.shimadzu.com/; MS, QTRAP® System, https://sciex.com/). The analytical conditions were as follows: UPLC: column, Waters ACQUITY UPLC HSS T3 C18 (1.8 *μ*m, 2.1 mm ∗ 100 mm); column temperature, 40°C; flow rate, 0.4 mL/min; injection volume, 2 *μ*L; solvent system, water (0.04% acetic acid): acetonitrile (0.04% acetic acid); gradient program, 95 : 5 V/V at 0 min, 5 : 95 V/V at 11.0 min, 5 : 95 V/V at 12.0 min, 95 : 5 V/V at 12.1 min, and 95 : 5 V/V at 14.0 min.

LIT and triple quadrupole (QQQ) scans were acquired on a triple quadrupole-linear ion trap mass spectrometer (QTRAP), QTRAP® LC-MS/MS System, equipped with an ESI Turbo Ion-Spray interface, operating in positive and negative ion modes and controlled by Analyst 1.6.3 software (Sciex). The ESI source operation parameters were as follows: source temperature 500°C; ion spray (IS) voltage 5500 V (positive) and −4500 V (negative); ion source gas I (GSI), gas II (GSII), and curtain gas (CUR) were set at 55, 60, and 25.0 psi, respectively; the collision induced dissociation (CAD) was high. Instrument tuning and mass calibration were performed with 10 and 100 *μ*mol/L polypropylene glycol solutions in QQQ and LIT modes, respectively. A specific set of MRM transitions were monitored for each period according to the metabolites eluted within this period.

### 2.8. Data Processing

For metabolic profile analysis, unsupervised principal component analysis (PCA) was performed by statistics function prcomp within R (http://www.r-project.org) and the data was unit variance scaled before unsupervised PCA. Orthogonal partial least-squares discriminant analysis (OPLS-DA) results were generated using R package MetaboAnalystR, from which we attained the variable importance in projection (VIP) values, score plots, and permutation plots. The data went under logarithmic transformation and mean centering before OPLS-DA, and to avoid overfitting, a permutation test (200 permutations) was performed. Hierarchical cluster analysis (HCA) results of samples and metabolites were presented as heatmap with dendrograms, while Pearson correlation coefficients (PCC) between samples were calculated by the cor function in R and presented as only heatmap. Both HCA and PCC were carried out by R package heatmap. For HCA, normalized signal intensities of metabolites (unit variance scaling) are visualized as a color spectrum. Receiver operating characteristic (ROC) curves were drawn by R (https://cran.r-project.org/web/packages/pROC/).

### 2.9. Biomarker Identification and Metabolic Pathway Analysis

VIP of the OPLS-DA model was preliminarily applied to screen out differential metabolites between the control group and the experimental group. Significantly regulated metabolites between groups were determined by VIP > 1, absolute Log2FC (fold change) ≥1, and FC ≤ 0.5. And if there was biological duplication in the sample grouping, based on the above, metabolites are selected with VIP>1 to be significantly different. The potential biomarkers were identified using Metware Database (MWDB). ROC was applied to analyze data for evaluating the predictive power of the identified biomarkers. Pathway analysis was based on the Kyoto Encyclopedia of Genes and Genomes (KEGG).

### 2.10. Statistical Analysis

Data were expressed as mean ± standard error of mean (SEM). The statistical analyses were performed using SPSS statistics software version 16.0 (SPSS Inc., Chicago, IL, USA). Comparison of the same parameter among groups was analyzed by one-way ANOVA followed by post hoc analysis with the Tukey test. *P* < 0.05 was considered statistically significant.

## 3. Results

### 3.1. Standardization of SQQX Extract

SQQX went through chemical standardization before being utilized as an intervention. An LC-MS approach was established to unveil the chemical profile of SQQX extract and quantify its main ingredients. Using different reference standard, 9 key compounds were identified from SQQX extract as listed in Supplementary Figure S1, and the minimum amount in mg/g of dried extract was 3.285526 betaine, 1.141584 for buddleoside, 0.409738 for chlorogenic acid, 0.333756 for chrysophanol, 0.203923 for aurantio-obtusin, 0.154462 for tanshinone IIA, 0.132823 for quercitrin, 0.0368 for rutinum, and 0.009707 for quercetin. The chemical analysis of SQQX extract here served as quality control for the reproducibility of the below animal study.

### 3.2. UPLC/MS Fingerprint and Its Repeatability

**Figure 1:**
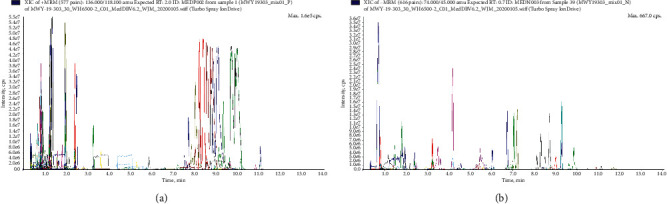
Representative positive (a) and negative (b) TIC of serum samples.

Both positive and negative ionization modes for serum metabolic profiling were used to analyze all 30 rat serum samples to get as many compounds as possible. Representative positive and negative TIC of serum obtained from the 5 groups is shown in Figure 1. The overlapping TIC diagrams with QC samples demonstrated the acceptable variations occurred during the large-scale sample analysis, and the results showed a dense distribution of the QC samples, which indicated the stability of the system and good quality of the data (Supplementary Figures S2a, S2b, and S3).

### 3.3. Changes of Rats in General Situation

**Figure 2:**
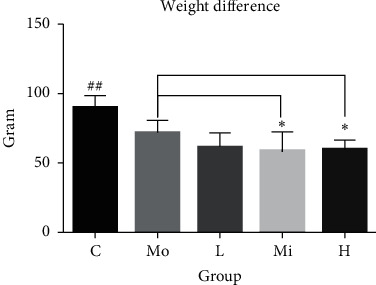
Comparison of weight difference in each group (^##^compared with all other groups, *P* < 0.001; ^*∗*^compared with the model group, *P* < 0.001).

Compared to the control group, the weight difference of the model group and the treatment groups were statistically significant (*P* < 0.001) (Supplementary Table 2). Besides, significant difference in body weight was found between the model group and the middle-dose and the high-dose SQQX groups, respectively (*P* < 0.001) (Figure 2). The rats in the model group were mentally very active, acted quickly, and were sensitive to stimuli such as sound. Additionally, they were more emotionally irritable, and often fought with each other or even bit people. Scratches or blood scabs could also be seen on some parts of their skin. Their hair was yellow and rough, with lack of luster, and they had hard stools. In contrast, rats in the control group were calmer in spirit, moderate in response to stimuli, and stable in mood and did less fight. They had soft, shiny hair and normal stools while rats in all three treatment groups were with reduced irritability, less fighting, improved hair color, and relatively soft stool in comparison to the model group.

### 3.4. BP Results

**Figure 3:**
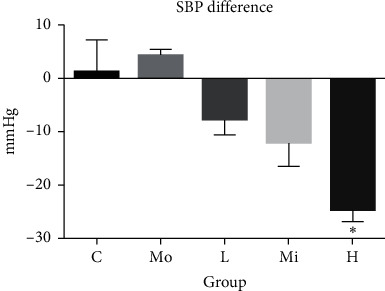
Comparison of SBP difference in each group (^*∗*^compared with the model group, *P* < 0.001).

Compared to the control group, the SBP of the model group was significantly increased (*P* < 0.001), which meant that the model was successful and stable (Table 1). And compared with the model group of the same period, the SBP of rats in the treatment groups was all decreased, and the difference was most statistically significant (*P* < 0.001) in the high-dose SQQX group. In addition, SBP difference of each group before and after intervention is shown in Figure 3.

### 3.5. Metabolic Profiles of the Control WKY and Model SHR

**Figure 4:**
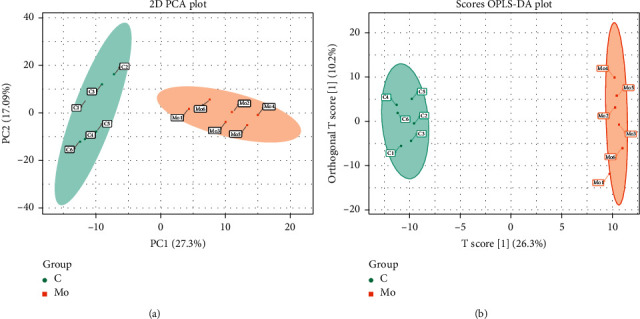
PCA and OPLS-DA score plots of the control and model groups: control group (green round); Mo: SHR group (orange round); PCA: principal component analysis. OPLS-DA: orthogonal partial least square discriminant analysis. Each point represented a subject.

To evaluate the alterations of metabolites in SHR, PCA and OPLS-DA were performed using data from the control and model groups. PCA score plots showed the obvious separation trend between the control and model group, indicating that serum metabolic states of hypertensive rats were significantly changed in relative to WKY rats. Moreover, OPLS-DA score plots were also separated into two clusters (Figure 4). These results illustrated that the metabolites in the control and model groups had been completely set apart from each other.

### 3.6. SQQX Altered Serum Metabolic Profiles of SHRs

**Figure 5:**
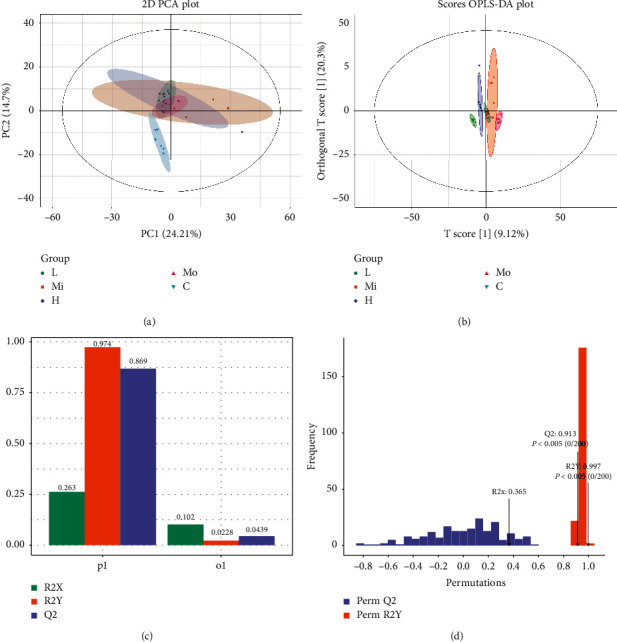
Alteration on the metabolic profiling by PCA and OPLS-DA with the SQQX intervention control group (blue triangle); Mo: SHR group (pink triangle); L: low-dose SQQX group (green round); M: middle-dose SQQX group (orange square); H: high-dose SQQX group (purple diamond) (a and b). Each point represented a subject. The OPLS-DA model and permutation test for OPLS-DA derived from the UPLC-MS/MS of serum obtained from the SHR group and high-dose SQQX groups (c and d). The R2Y value represented the goodness of the model, and the Q2 value represented the predictability of the models. The closer R2Y and Q2 to 1, the better.

As to unfold the effect of SQQX on metabolic profiles in hypertensive rats, OPLS-DA analysis was performed to obtain the changes in metabolic trajectory. The OPLS-DA score plot showed that the five groups of serum samples were obviously divided into 5 categories, and the metabolic trajectory of the high-dose SQQX group deviated from the model group and moved to the control group, which indicated that after SQQX treatment, serum metabolites were obviously changed and were inclined to recover. Moreover, the high-dose SQQX group seemed to have the best efficacy among all three intervention groups (Figure 5).

### 3.7. Pathways Related to SQQX Intervention

**Figure 6:**
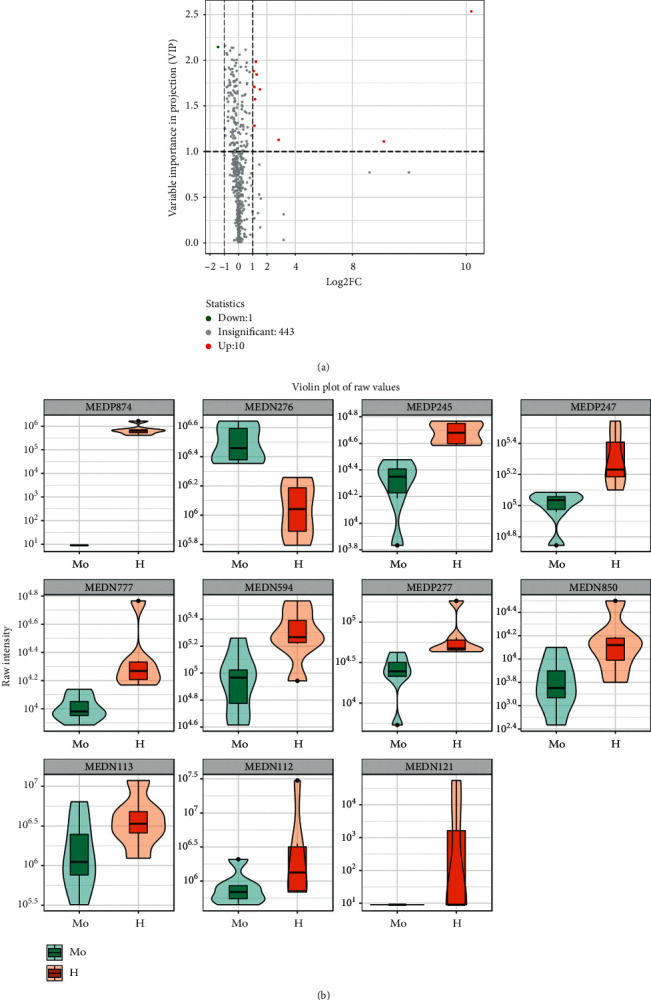
The relative content of the 11 potential biomarkers: (a) upregulated (red dots) and downregulated (green dots); (b) model group (green) and high-dose SQQX group (orange).

**Figure 7:**
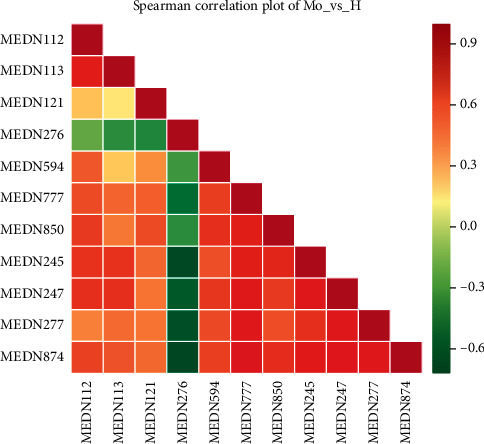
Correlation of metabolites in the high-dose SQQX group compared with the model.

A total of 11 specific biomarkers were identified in the high-dose SQQX group compared to the model group (Table 2), in which 10 metabolites were upregulated while 1 was downregulated (Figure 6). And correlation of these metabolites was presented in a heatmap (Figure 7).

**Figure 8:**
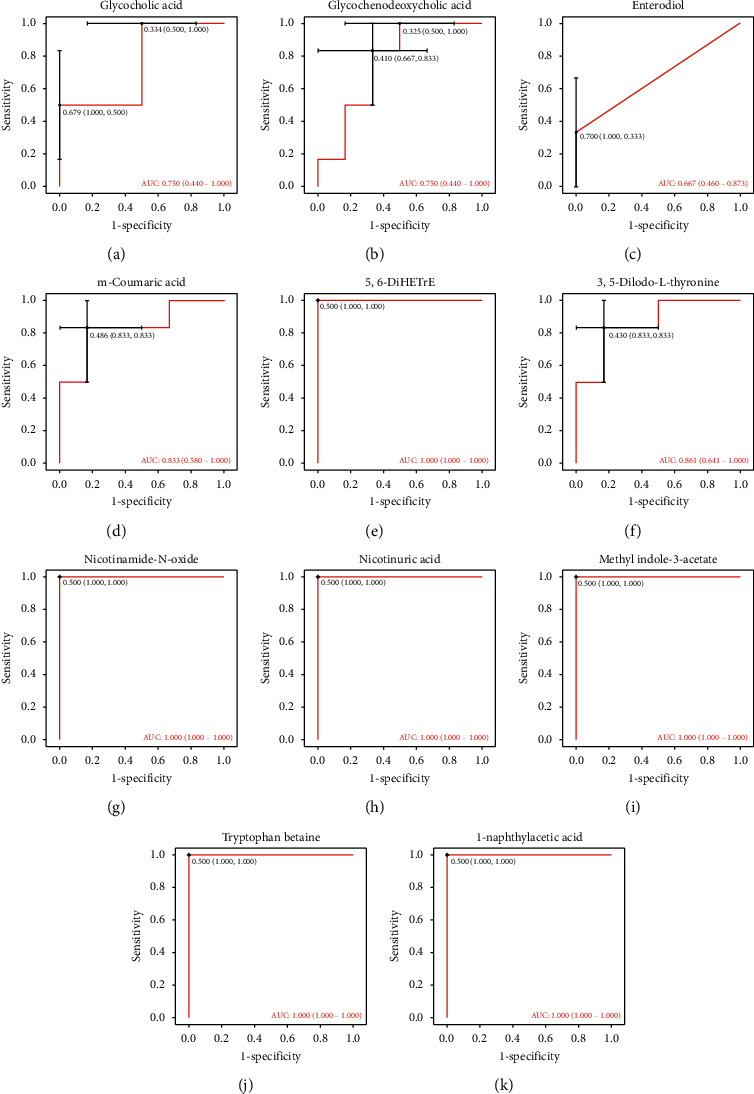
AUC of the 11 metabolites after high-dose SQQX intervention.

Additionally, ROC curves were performed to access the predictive value of these screened potential biomarkers. The 10 upregulated metabolites showed good diagnostic ability with average area under the curve (AUC) at 0.667∼1, and the 1 downregulated metabolite also displayed good predictive capability for diagnosis with AUC at 1 (Figure 8).

**Figure 9:**
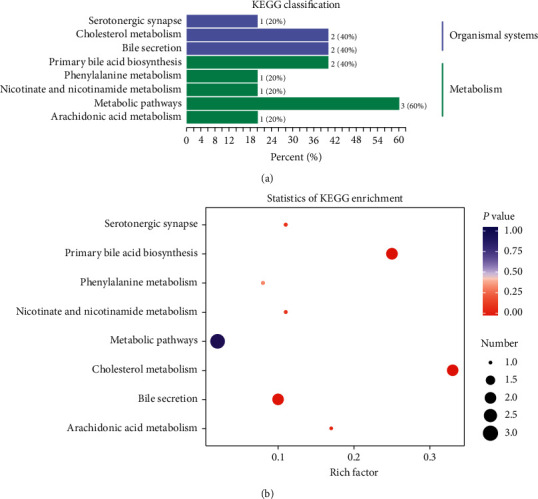
Pathway related to high-dose SQQX treatment.

Moreover, pathway analysis demonstrated that metabolites that might mark protective effects of SQQX against HTN were glycocholic acid, glycochenodeoxycholic acid, nicotinamide-N-oxide, tryptophan betaine, etc., involving in many metabolic pathways, such as cholesterol metabolism, primary bile acid biosynthesis, bile secretion, and nicotinate and nicotinamide metabolism (Figures 9(a) and 9(b)).

## 4. Discussion

This study was the first metabolomics-based analysis of SQQX's effect on SHR using HPLC/MS approach. SQQX markedly improved general condition of SHR, reduced SBP and altered serum metabolites, indicating that it could somehow regulate the perturbed metabolism in SHR.

HTN is a severe world health threat with high incidence and high mortality but low control rate, causing a huge burden to the global society. Yet, due to effects of multiple genes and characteristics of heterogeneity, the pathological mechanism of HTN and effects of antihypertensive drugs have not been fully elucidated. Metabolomics studies metabolic network changes of organisms from an overall perspective and links metabolites with biological processes to reveal changes of organisms induced by environmental stimuli, from which we could know what has exactly happened in vivo [9, 15]. Thus, it offers a strong and useful way to explore the pathogenesis and new treatments of HTN. TCM has been of great therapeutic effect on HTN; however, the inherent working network is too complex to explain merely from a specific target or signaling pathway and that is where metabolomics could shed light on and work in correspondence with the integral view of TCM. In recent years, many researchers have applied metabolomics to study HTN in search of the pathogenesis, potential biomarkers, the impact of lifestyle and drug intervention, and the effect mechanism in a person or animal model by identifying and comparing metabolites in blood, urine, or tissues [13, 16–18].

In the present study, we identified 11 significant metabolites regarding SQQX's intervention on SHRs compared to the model group. Glycosylcholic acid, glycosyldeoxycholic acid, nicotinamide-N-oxide, and glycine-metabolism-related products were upregulated, indicating that SQQX displayed a regulatory effect on serum metabolites which mainly related to bile acid and coenzyme metabolism including GSH, NADH, and NADPH. Nicotinamide is an important precursor of NADPH and NADH, which play a key role in REDOX balance. Studies have shown that reactive oxygen species (ROS) derived from NADPH oxidase (NOX) play an important role in the occurrence and development of vascular remodeling in HTN. In both SHR and angiotensin II (AngII) induced hypertensive models, the expression and activity of NOX subunits were significantly increased, and ROS surged. Inhibition of NOX could reduce the production of O_2_^−^ in the vascular wall and prevent progression of HTN [19]. Apart from this, 8-week supplementation of niacin (precursor of NAD) increased the content of niacin in the cell membrane and decreased the concentration of Na+ in the cytoplasm as well as SBP in hypertensive patients, suggesting that niacin may lower BP by changing the activity of the cell membrane sodium transport system [20].

Furthermore, primary bile acid biosynthesis, bile secretion, and cholesterol metabolism are three major pathways concerning SQQX intervention based on KEGG analysis. According to earlier research, HTN and lipid metabolic disorders often coexist as a result of their common metabolic and genetic backgrounds. They interact with each other via a variety of mechanisms, sharing a mutually interdependent relationship and working together in aggravating HTN. Statistics showed that more than 56% of hypertensive patients had dyslipidemia. As predisposing factors for AS, also a nonnegligible process in the development of cardiovascular diseases, HTN and dyslipidemia jointly promote target organ damage such as coronary heart disease, stroke, kidney damage, and large artery disease [20]. Clinical and epidemiological studies confirmed that blood total cholesterol (TC) levels were positively correlated with BP [21]. People with high baseline TC, high TC, or high-density lipoprotein cholesterol (HDLC) ratios were more likely to have HTN [22]. Castelli and Anderson found in the Framingham Heart Study that patients with HTN had higher TC levels, and there was a strong positive correlation between BP and TC levels (*r* = 0.12) [23]. Bonaa and Thelle analyzed the relation among TC, HDLC, non-high-density lipoprotein cholesterol (nHDLC), triacylglycerol (TG) level, and BP in 8081 men aged 20 to 54 years and 7663 women aged 20 to 49 years [24]. Results showed that the levels of TG and nHDLC of both genders increased significantly with the surge of SBP and diastolic blood pressure (DBP) and blood TC levels were independently positively associated with DBP and SBP. However, the mechanism remains unclear. Most scholars have perceived abnormal endothelial function, aberrant vascular reactivity, and cell membrane damage as important mechanisms of lipid abnormalities causing HTN. Oxidized low-density lipoprotein (OX-LDL) pathway could reduce the release of nitric oxide (NO) vascular ECs and prostaglandin I2 (PGI2) and increase levels of vasoconstrictors such as endothelin 1 (ET-1) and thromboxane A2 (TXA2), which reduces the elasticity and compliance of blood vessels and directly leads to the increase in BP [25]. Meanwhile, the effect of Ang II in raising BP was largely affected by plasma cholesterol levels, especially the LDL (low-density lipoprotein) [26]. LDL was positively related to both SBP (*r* = 0.24) and DBP increase (*r* = 0.23) after AngII injection. In addition, changes in blood TC, TG, and phospholipids could affect the lipid composition of cell membranes, which in turn influence the physical and chemical properties of cell membranes, such as membrane fluidity and ion channels. Serum TC, TG, and LDL in hypertensive patients could all act on cell membranes to change their permeability and have been found a positively correlated relationship with cellular Ca^2+^ influx. The increase of Ca^2+^ influx leads to increased contraction of vascular smooth muscle (VSM) and peripheral vascular resistance, resulting in high BP [27].

Bile acids are the final product of cholesterol metabolism. The conversion of cholesterol into bile acid is the main way to eliminate cholesterol in the human body. Cholic acid and chenodeoxycholic acid are the two main types of primary bile acids synthesized. They can be combined with glycine, taurine, etc. to form bound bile acids, and after being discharged into the intestinal cavity, they get decomposed into deoxycholic acid and lithocholic acid by the bacteria in the ileum and colon, playing a role in promoting the absorption of fat-soluble cellulose and dietary fat [28]. In 1932, Meakins firstly put forward the notion of “catarrhal jaundice” for patients with HTN since he found that after the onset of jaundice, the BP of hypertensive patients quickly returned to normal, and the BP did not rise again until long after the jaundice disappeared [29]. Later, scholars have discovered that bile acid is an endogenous vasodilator, and intravenous injection of bile acid in animals is able to cause hypotension [28]. Tominaga et al. also proved that bile acids could bring down BP in SHR and concentration-dependently weaken the responsiveness of peripheral blood vessels to norepinephrine (NE). Moreover, bile acids with high hydrophobicity, such as deoxycholic acid, were less reactive to NE than those with low hydrophobicity such as taurocholic acid [30, 31]. Levy et al. revealed that bile acids might change the sensitivity of the renal vascular system to various vasodilator factors by affecting second messengers of cGMP such as bradykinin and acetylcholine, and the latter could induce endothelial cells (ECs) to release NO, which activated guanylate cyclase, relaxed VSM, and led to a drop in BP [32]. Additionally, it is well known that the concentration of free calcium in cells determines the tension of blood vessels, and the regulation of free calcium in cells depends on calcium transport and binding processes on the plasma membrane. Researchers have found that although bile acids could not directly pass through the cell membrane of VSM cells, they were able to act as a Ca^2+^ carrier, promoting Ca^2+^ uptake in liver cells, increasing Ca^2+^ influx, and participating in the regulation of vascular wall tension [33, 34]. In this study, the serum levels of glycocholic acid and glycodeoxycholic acid in rats increased significantly after the treatment of high-dose SQQX, indicating that SQQX may regulate BP of SHR by getting involved in the process of converting cholesterol into bile acid, promoting the synthesis of primary and secondary bile acid, prompting absorption of cholesterol to reduce serum cholesterol level.

Our previous research demonstrated that SQQX could effectively and smoothly reduce both the SBP and DBP of hypertensive patients. Moreover, it could decline the levels of serum TC, LDLC, and TG while elevating HDLC (*P* < 0.01) and flow mediated dilation (FMD) parameters of patients, improving their vascular endothelial function [35, 36]. One of the studies of 78 HTN patients with liver and kidney yin deficiency and phlegm turbidity found that SBP (*P* < 0.001) and DBP (*P* < 0.05) were significantly reduced in the SQQX treatment group after 4 weeks with an efficient of 90.6%. Meanwhile, serum ET was markedly reduced (*P* < 0.001) while calcitonin gene-related peptide and SOD were in a rise [35]. TC and LDLC were reduced from 5.37 ± 1.40 to 4.46 ± 0.42 mmol/L (*P* < 0.001) and 3.06 ± 0.76 to 2.65 ± 0.65 mmol/L (*P* < 0.05) after treatment while HDLC was raised up. Another study found that after 8-week treatment with SQQX, the total decrease in SBP and DBP was 10.64 mmHg and 6.64 mmHg (*P* < 0.01) and the average SBP at the 24 h morning peak declined while the valley-to-peak ratio escalated in HTN patients (both *P* < 0.05). FMD increased evidently with an absolute value of 2.16 (*P* < 0.01). TC, LDLC, TG, and HDLC were also improved by SQQX [36]. A recent study further revealed that PPARs, core module in regulating lipid synthesis and metabolism, were remarkably increased with SQQX intervention, which may be related to reduction in BP reduction and improvement of myocardial fibrosis [7]. These results were consistent with the decrease in the level of harmful oxidative lipoproteins observed in the present study, and the increase in the level of 5(6)-DiHET, which was conducive to the production of the vasodilation factor PGE1. We hypothesized that reducing blood lipids would be one of the key mechanisms regarding SQQX's effect on lowering BP.

To sum up, cholesterol metabolism, bile acid biosynthesis and metabolism, and nicotinate and nicotinamide metabolism are major pathways correlated to SQQX's effects on SHR. SQQX may reduce BP of SHR and exert protective effects by regulating bile acid and coenzyme metabolism to prevent vascular inflammation and oxidative stress process. Nevertheless, due to an approach of broad-spectrum targeted HPLC-based metabolic analysis which is ideal for detecting medium-polar and high-polar compounds comprising fatty acids, phenols, vitamins, organic acids, nucleotides, flavonoids, lipids, etc., the current study may have the limitation of not being able to detect some of the metabolites in SHR serum, especially those easily volatile and heat-resistant products. Also, individual difference of SHR models may be a factor that heavily influences final outcome. Owing to economic reasons, we have got the minimum number of 6 rats in each group to meet a statistical criteria and results would be more reliable with larger sample size to minimize systemic errors and confounding bias. Besides, since orally taken drugs usually go through blood absorption and urine excretion, it would be better if both serum and urine samples are collected for metabolite detection and comparisons are then made to figure out more precise small metabolite change within the organism. Further comprehensive study is still needed to illustrate the detailed action mechanism and regulating network of SQQX's effect on HTN.

## 5. Conclusion

This study was the first to apply broad-spectrum targeted metabolomic approach using the UPLC/MS method to investigate the protective effects of SQQX against HTN in SHR. A total of 11 metabolites contributing to SQQX's efficacy on high BP were identified, and main pathways related were primary bile acid biosynthesis, cholesterol metabolism, bile secretion, and metabolic pathways. Treatments with SQQX attenuated hypertensive injury partially by altering serum metabolomics, and the high-dose SQQX seemed the best.

## Figures and Tables

**Table 1:** SBP of each group expressed in mean ± SEM.

Group	0 day (mmHg)	90 days (mmHg)	SBP difference
Control group (C)	176.33 ± 5.54	177.50 ± 2.13	1.17 ± 5.98
Model group (Mo)	233.83 ± 3.42^#^	238.00 ± 2.57^#^	4.17 ± 1.25
Low-dose SQQX group (L)	237.17 ± 2.10	229.50 ± 3.68	−7.67 ± 2.93
Middle-dose SQQX group (Mi)	235.00 ± 1.18	223.00 ± 4.68	−12.00 ± 4.47
High-dose SQQX group (H)	235.83 ± 1.52	211.33 ± 3.58^*∗*^	−24.50 ± 2.32^*∗*^

^#^Compared with the control group, *P* < 0.001; ^*∗*^compared with the model group, *P* < 0.001.

**Table 2:** Differential metabolites in the high-dose SQQX group.

No.	Abbreviation	Molecular formula	VIP	Log2FC	Trend
MEDN112	Glycocholic acid	C_26_H_43_NO_6_	1.13	2.82	Up
MEDN113	Glycochenodeoxycholic acid	C_26_H_43_NO_5_	1.28	1.12	Up
MEDN121	Enterodiol	C_18_H_22_O_4_	1.11	10.21	Up
MEDN276	1-Naphthylacetic acid	C_12_H_10_O_2_	2.14	−1.43	Down
MEDN594	*m*-Coumaric acid	C_9_H_8_O_3_	1.71	1.11	Up
MEDN777	5,6-DiHETrE	C20H34O4	1.85	1.27	Up
MEDN850	3,5-Diiodo-L-thyronine	C_15_H_13_I_2_NO_4_	1.57	1.16	Up
MEDP245	Nicotinamide-N-oxide	C_6_H_6_N_2_O_2_	1.98	1.22	Up
MEDP247	Nicotinuric acid	C_8_H_8_N_2_O_3_	1.88	1.07	Up
MEDP277	Methyl indole-3-acetate	C_11_H_11_NO_2_	1.68	1.50	Up
MEDP874	Tryptophan betaine	C_14_H_18_N_2_O_2_	2.53	16.37	Up

## Data Availability

The data used to support findings of this study are available by requests to the corresponding author.

## Supplementary Materials

Supplementary Materials Table S1: the herbal composition of SQXX. Table S2: weight of each group expressed in mean ± SEM. ^#^Compared to the control group, *P* < 0.001; ^*∗*^compared with the model group, *P* < 0.001. Table S3: content of main compounds found in SQQX. Figure S1: total iron current chromatograms of SQQX, (a) positive mode; (b) negative mode. RT: retention time. Figure S2: overlapping TIC diagrams with QC samples in negative mode (a) and positive mode (b). Figure S3. PCA score plots of serum samples and QC samples in positive and negative ion modes. Figure S4. Z score plot of biomarkers identified in the model group and high-dose SQQX group.

## References

[1] Mensah G. A. (2016). Hypertension and target organ damage: don’t believe everything you think!. *Ethnicity & Disease*.

[2] Kearney P. M., Whelton M., Reynolds K., Muntner P., Whelton P. K., He J. (2005). Global burden of hypertension: analysis of worldwide data. *The Lancet*.

[3] Hu S. S. G. R. (2019). Summary of the 2018 report on cardiovascular diseases in China. *Chinese Circulation Journal*.

[4] Forouzanfar M. H., Forouzanfar M. H., Alexander L. (2015). Global, regional, and national comparative risk assessment of 79 behavioural, environmental and occupational, and metabolic risks or clusters of risks in 188 countries, 1990–2013: a systematic analysis for the global burden of disease study 2013. *Lancet (London, England)*.

[5] Mills K. T., Bundy J. D., Kelly T. N. (2016). Global disparities of hypertension prevalence and control: a systematic analysis of population-based studies from 90 countries. *Circulation*.

[6] Li L. (2006). *Research on Influences of Jiangyamaijing Solution on Inflammatory Factors and Left Ventricular Remodeling in Rat with Spontaneously Hypertension*.

[7] Chen L. Y., Pan C. S., Wei X. H., Li L., Han J. Y., Huang L. (2013). Sang-qi granula reduces blood pressure and myocardial fibrosis by suppressing inflammatory responses associated with the peroxisome proliferator-activated receptors and nuclear factor kappa B protein in spontaneously hypertensive rats. *Evidence-Based Complementary and Alternative Medicine*.

[8] Li L. F. R. H. (2015). Effects of sangqi qingxuan granule on cardiomyocyte apoptosis in spontaneously hypertensive rats. *Chinese Journal of Integrative Medicine on Cardio-/Cerebrovascuiar Disease*.

[9] Johnson C. H., Ivanisevic J., Siuzdak G. (2016). Metabolomics: beyond biomarkers and towards mechanisms. *Nature Reviews Molecular Cell Biology*.

[10] Wang M., Chen L., Liu D., Chen H., Tang D.-D., Zhao Y.-Y. (2017). Metabolomics highlights pharmacological bioactivity and biochemical mechanism of traditional Chinese medicine. *Chemico-Biological Interactions*.

[11] Jiang H., Shen Z., Chu Y. (2015). Serum metabolomics research of the anti-hypertensive effects of Tengfu Jiangya tablet on spontaneously hypertensive rats. *Journal of Chromatography B*.

[12] Yang M., Yu Z., Deng S. (2016). A targeted metabolomics MRM-MS study on identifying potential hypertension biomarkers in human plasma and evaluating acupuncture effects. *Scientific Reports*.

[13] Liu A., Chu Y. J., Wang X. (2018). Serum metabolomics study based on LC-MS and antihypertensive effect of uncaria on spontaneously hypertensive rats. *Evidence-Based Complementary and Alternative Medicine*.

[14] Dong H., Zhang S., Du W., Cong H., Zhang L. (2020). Pharmacodynamics and metabonomics study of Tianma Gouteng decoction for treatment of spontaneously hypertensive rats with liver-yang hyperactivity syndrome. *Journal of Ethnopharmacology*.

[15] Zhang A., Sun H., Wang X. (2012). Serum metabolomics as a novel diagnostic approach for disease: a systematic review. *Analytical and Bioanalytical Chemistry*.

[16] Yang M., Lao L. (2019). Emerging applications of metabolomics in traditional Chinese medicine treating hypertension: biomarkers, pathways and more. *Frontiers in Pharmacology*.

[17] Lin Y. T., Salihovic S., Fall T. (2020). Global plasma metabolomics to identify potential biomarkers of blood pressure progression. *Arteriosclerosis, Thrombosis, and Vascular Biology*.

[18] Santiago-Hernandez A., Martinez P. J., Martin-Lorenzo M. (2020). Differential metabolic profile associated with the condition of normoalbuminuria in the hypertensive population. *Nefrologia*.

[19] Ge L. Q. S. D. (2013). NADPH oxidase family and vascular remodeling in hypertension. *Journal of Clinical and Pathological Research*.

[20] Peng H. S. Q. (2007). Association of dyslipidemia and hypertension. *Chinese Journal of Hypertension*.

[21] Goode G. K., Miller J. P., Heagerty A. M. (1995). Hyperlipidaemia, hypertension, and coronary heart disease. *The Lancet*.

[22] Zavaroni I., Bonora E., Pagliara M. (1989). Risk factors for coronary artery disease in healthy persons with hyperinsulinemia and normal glucose tolerance. *New England Journal of Medicine*.

[23] Castelli W. P., Anderson K. (1986). A population at risk: prevalence of high cholesterol levels in hypertensive patients in the framingham study. *The American Journal of Medicine*.

[24] Bønaa K. H., Thelle D. S. (1991). Association between blood pressure and serum lipids in a population. The tromsø study. *Circulation*.

[25] Chin J. H., Azhar S., Hoffman B. B. (1992). Inactivation of endothelial derived relaxing factor by oxidized lipoproteins. *Journal of Clinical Investigation*.

[26] Vuagnat A., Giacché M., Hopkins P. N. (2001). Blood pressure response to angiotensin II, low-density lipoprotein cholesterol and polymorphisms of the angiotensin II type 1 receptor gene in hypertensive sibling pairs. *Journal of Molecular Medicine*.

[27] Tan G. (1997). Relationship between plasma lipids and membrane ion transport in hypertensive patients. *Journal of Third Military Medical University*.

[28] Bomzon A., Ljubuncic P. (1995). Bile acids as endogenous vasodilators?. *Biochemical Pharmacology*.

[29] Meakins J. C. (1932). Jaundice and blood pressure. *Medical Clinics of North America*.

[30] Tominaga T., Suzuki H., Ogata Y., Imafuku T., Saruta T. (1988). Bile acids are able to reduce blood pressure by attenuating the vascular reactivity in spontaneously hypertensive rats. *Life Sciences*.

[31] Bomzon A., Finberg J. P. M., Tovbin D., Naidu S. G., Better O. S. (1984). Bile salts, hypotension and obstructive jaundice. *Clinical Science*.

[32] Levy M., Finestone H., Fechner C. (1984). Action of renal vasodilators in dogs following acute biliary obstruction. *Journal of Surgical Research*.

[33] Beuers U., Nathanson M. H., Boyer J. L. (1993). Effects of tauroursodeoxycholic acid on cytosolic Ca^2+^ signals in isolated rat hepatocytes. *Gastroenterology*.

[34] Thibault N., Ballet F. (1993). Effect of bile acids on intracellular calcium in isolated rat hepatocyte couplets. *Biochemical Pharmacology*.

[35] Huang L. (2006). *Clinical and Experimental Study on the Effect of Jiangyamaijing Liquid on Hypertension and Hypertensive Myocardial Fibrosis*.

[36] Li Y. J. (2015). *Clinical Observation of Sangqiqingxuan Granule in the Treatment of Essential Hypertension and its Effect on Vascular Endothelial Function*.

