# Supplementary material for: Mechanism of SQQX Decoction's Protective Effect on SHR: A Serum Metabolomics-Based Analysis
Source: Evid Based Complement Alternat Med. 2020 Dec 9;2020:8856943. doi: 10.1155/2020/8856943 (PMC7744199; doi:10.1155/2020/8856943)
Supplement: Supplementary Materials — Table S1: the herbal composition of SQXX. Table S2: weight of each group expressed in mean ± SEM. #Compared to the control group, P < 0.001; ∗compared with the model group, P < 0.001. Table S3: content of main compounds found in SQQX. Figure S1: total iron current chromatograms of SQQX, (a) positive mode; (b) negative mode. RT: retention time. Figure S2: overlapping TIC diagrams with QC samples in negative mode (a) and positive mode (b). Figure S3. PCA score plots of serum samples and QC samples in positive and negative ion modes. Figure S4. Z score plot of biomarkers identified in the model group and high-dose SQQX group. [file 8856943.f1.zip › Supplementary/Supplementary Table.S3 Content of Main Compounds Found in SQQX.docx]

**Table.3 Content of Main Compounds Found in SQQX**

| Component | Content(mg/g) |
| --- | --- |
| Betaine | 3.285526 |
| Buddleoside | 1.141584 |
| chlorogenic acid | 0.409738 |
| Chrysophanol | 0.333756 |
| aurantio-obtusin | 0.203923 |
| tanshinone IIA | 0.154462 |
| Quercitrin | 0.132823 |
| rutinum | 0.0368 |
| quercetin | 0.009707 |
