# Supplementary figures and images for: Mechanism of SQQX Decoction's Protective Effect on SHR: A Serum Metabolomics-Based Analysis
Source: Evid Based Complement Alternat Med. 2020 Dec 9;2020:8856943. doi: 10.1155/2020/8856943 (PMC7744199; doi:10.1155/2020/8856943)

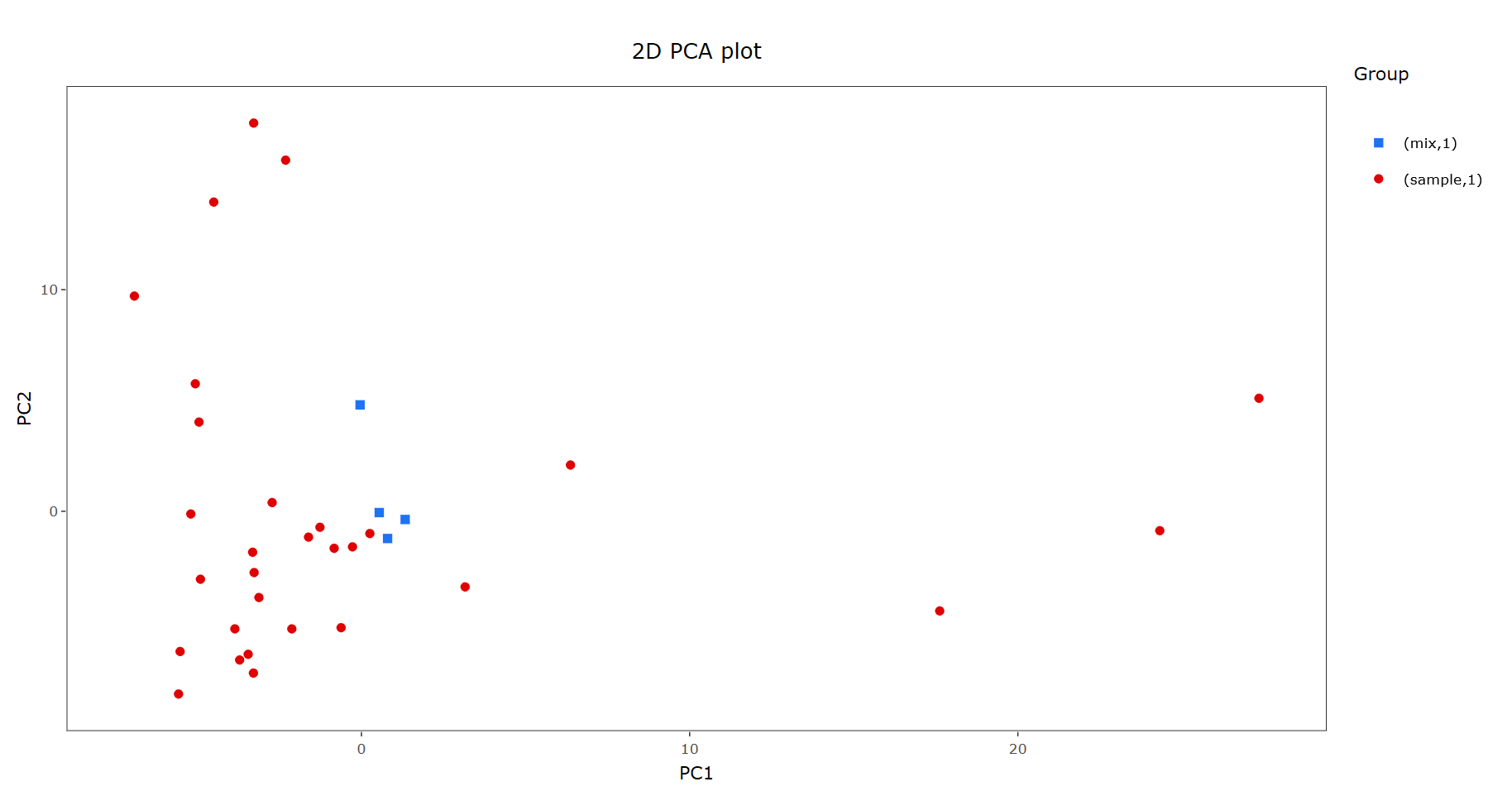


1. Negative mode


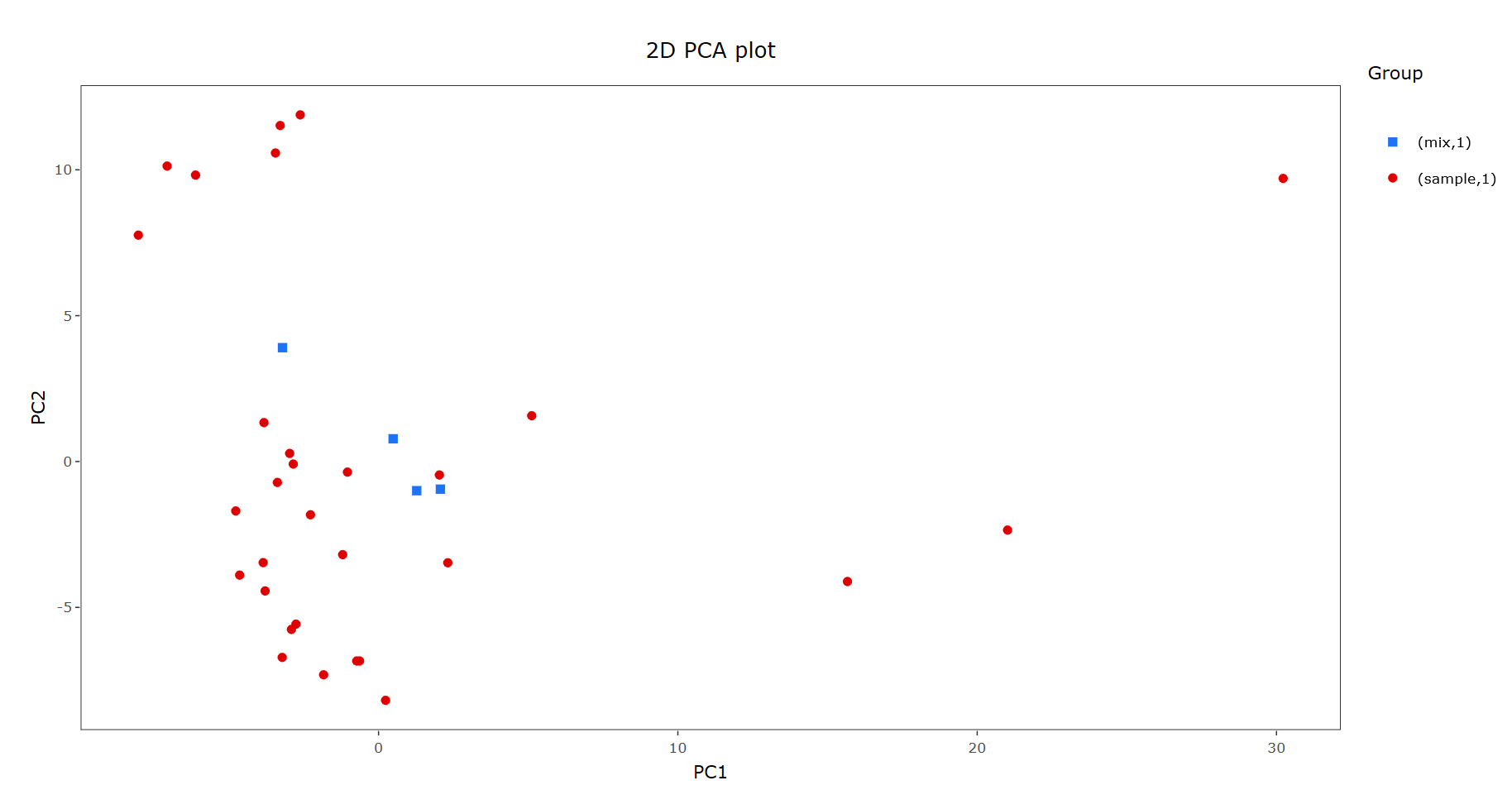


1. Possitive mode

Supplement: Supplementary Materials — Table S1: the herbal composition of SQXX. Table S2: weight of each group expressed in mean ± SEM. #Compared to the control group, P < 0.001; ∗compared with the model group, P < 0.001. Table S3: content of main compounds found in SQQX. Figure S1: total iron current chromatograms of SQQX, (a) positive mode; (b) negative mode. RT: retention time. Figure S2: overlapping TIC diagrams with QC samples in negative mode (a) and positive mode (b). Figure S3. PCA score plots of serum samples and QC samples in positive and negative ion modes. Figure S4. Z score plot of biomarkers identified in the model group and high-dose SQQX group. [file 8856943.f1.zip › Supplementary/Supplementary Figure S3. PCA score plots of serum samples and QC samples in positive and negative ion modes.docx]

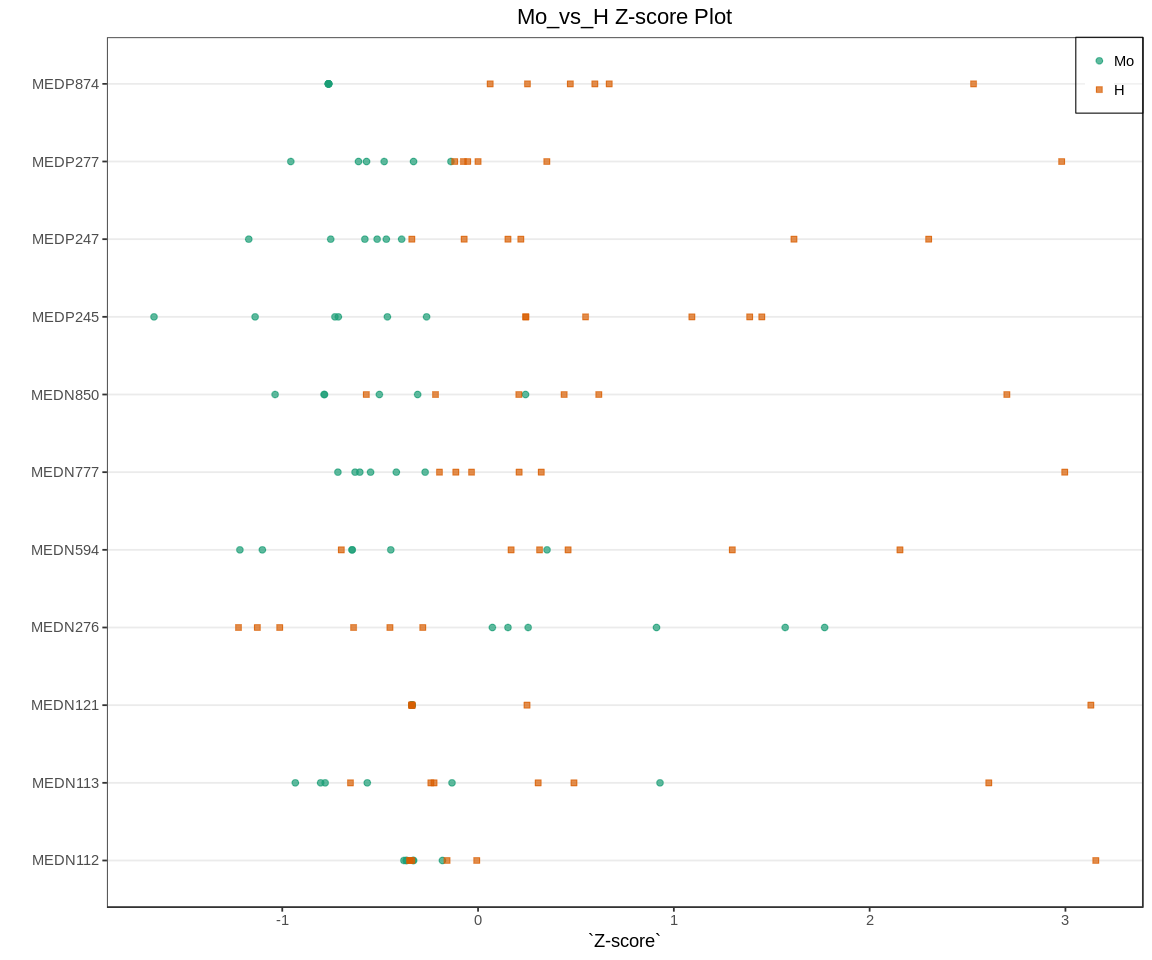

Supplement: Supplementary Materials — Table S1: the herbal composition of SQXX. Table S2: weight of each group expressed in mean ± SEM. #Compared to the control group, P < 0.001; ∗compared with the model group, P < 0.001. Table S3: content of main compounds found in SQQX. Figure S1: total iron current chromatograms of SQQX, (a) positive mode; (b) negative mode. RT: retention time. Figure S2: overlapping TIC diagrams with QC samples in negative mode (a) and positive mode (b). Figure S3. PCA score plots of serum samples and QC samples in positive and negative ion modes. Figure S4. Z score plot of biomarkers identified in the model group and high-dose SQQX group. [file 8856943.f1.zip › Supplementary/Supplementary Figure S4. Mo_vs_H_Z Score.tif]

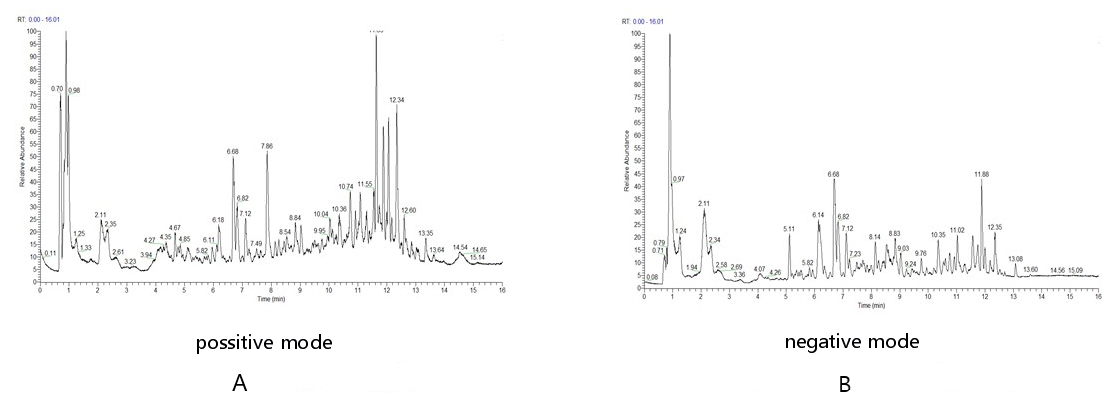

Supplement: Supplementary Materials — Table S1: the herbal composition of SQXX. Table S2: weight of each group expressed in mean ± SEM. #Compared to the control group, P < 0.001; ∗compared with the model group, P < 0.001. Table S3: content of main compounds found in SQQX. Figure S1: total iron current chromatograms of SQQX, (a) positive mode; (b) negative mode. RT: retention time. Figure S2: overlapping TIC diagrams with QC samples in negative mode (a) and positive mode (b). Figure S3. PCA score plots of serum samples and QC samples in positive and negative ion modes. Figure S4. Z score plot of biomarkers identified in the model group and high-dose SQQX group. [file 8856943.f1.zip › Supplementary/Supplementary Figure.S1 Total iron current chromatograms of SQQX .tif]

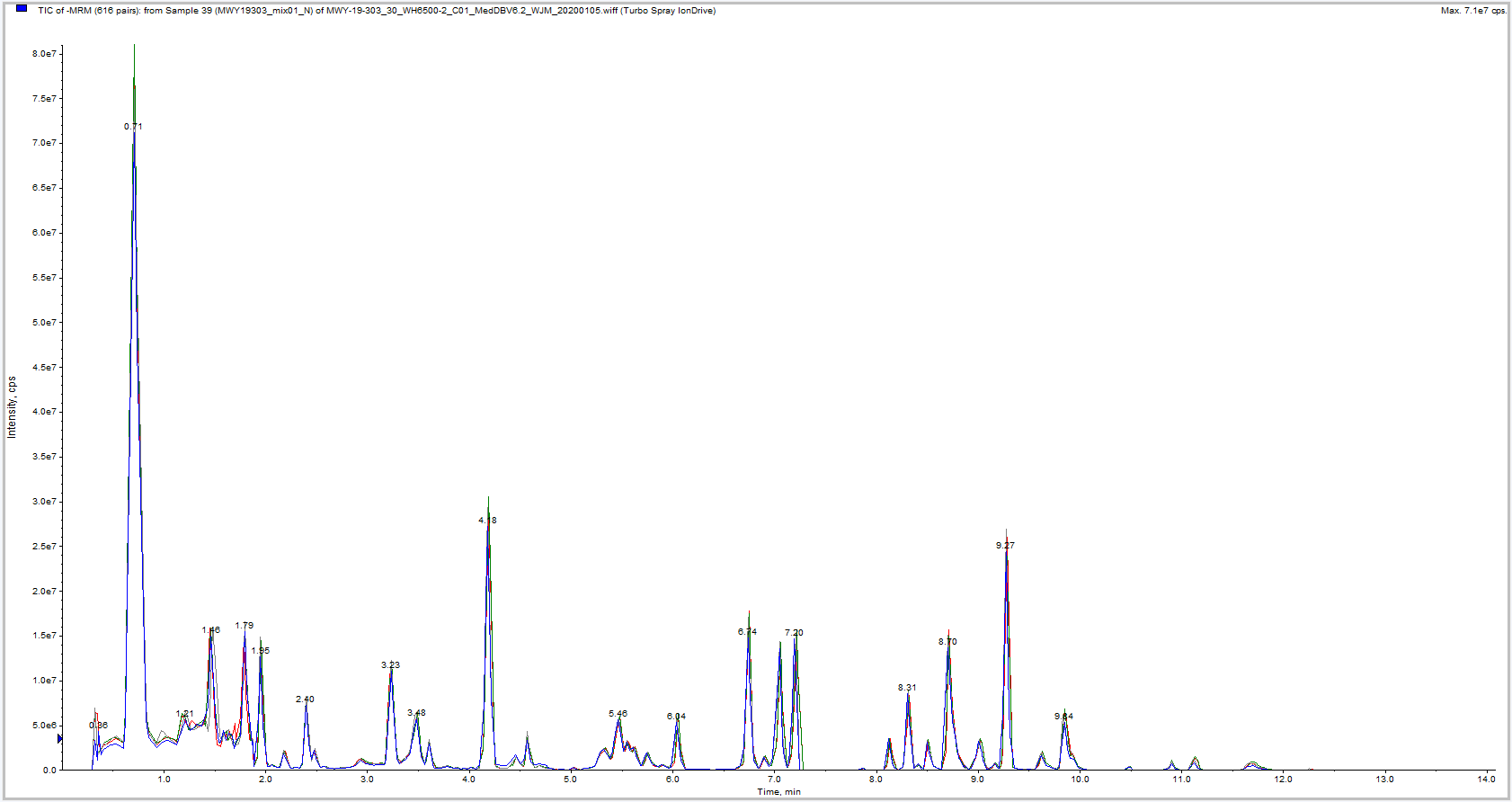

Supplement: Supplementary Materials — Table S1: the herbal composition of SQXX. Table S2: weight of each group expressed in mean ± SEM. #Compared to the control group, P < 0.001; ∗compared with the model group, P < 0.001. Table S3: content of main compounds found in SQQX. Figure S1: total iron current chromatograms of SQQX, (a) positive mode; (b) negative mode. RT: retention time. Figure S2: overlapping TIC diagrams with QC samples in negative mode (a) and positive mode (b). Figure S3. PCA score plots of serum samples and QC samples in positive and negative ion modes. Figure S4. Z score plot of biomarkers identified in the model group and high-dose SQQX group. [file 8856943.f1.zip › Supplementary/Supplementary Figure.S2a. Overlapping TIC diagrams with QC samples-negative mode.tif]

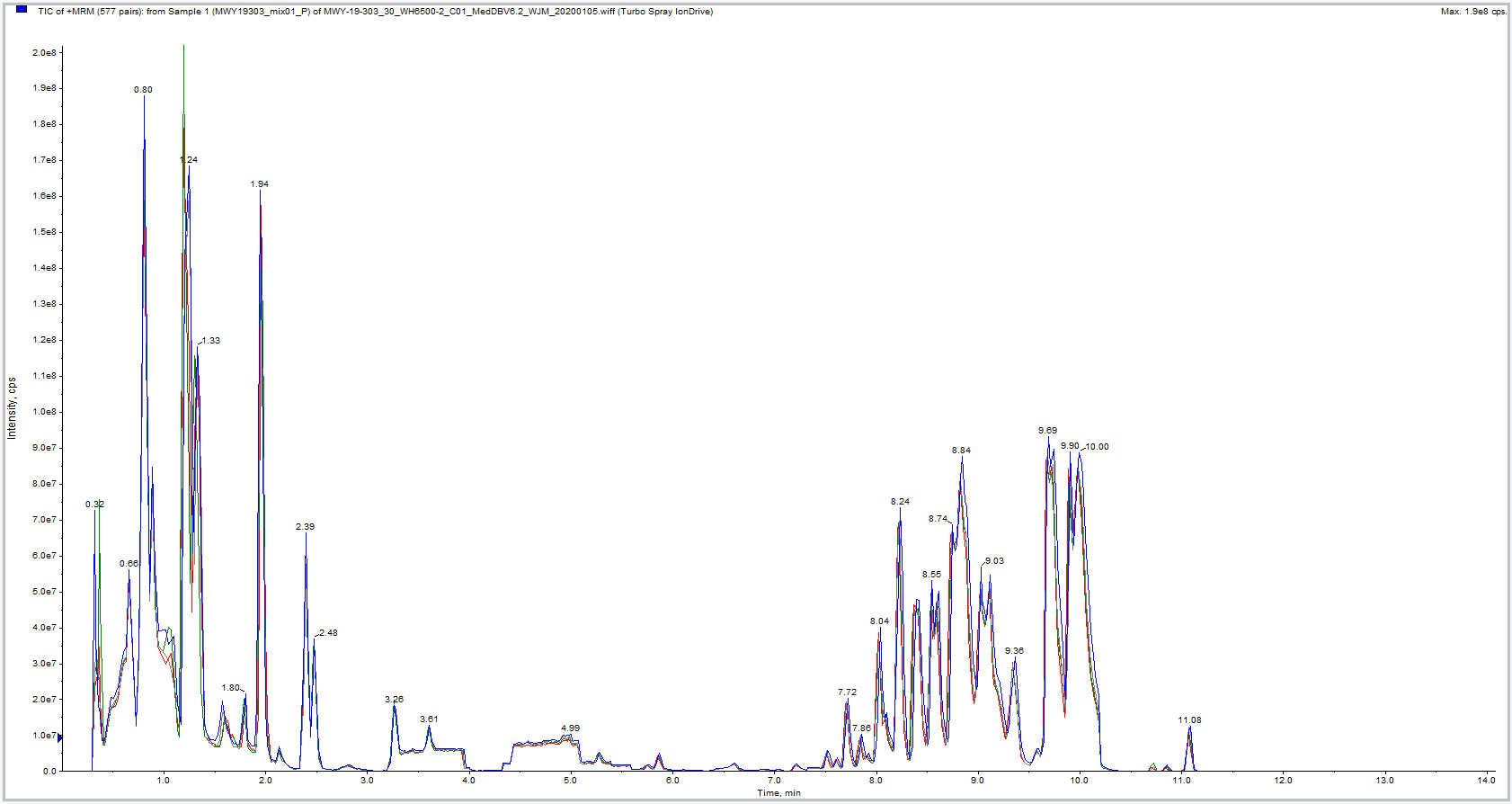

Supplement: Supplementary Materials — Table S1: the herbal composition of SQXX. Table S2: weight of each group expressed in mean ± SEM. #Compared to the control group, P < 0.001; ∗compared with the model group, P < 0.001. Table S3: content of main compounds found in SQQX. Figure S1: total iron current chromatograms of SQQX, (a) positive mode; (b) negative mode. RT: retention time. Figure S2: overlapping TIC diagrams with QC samples in negative mode (a) and positive mode (b). Figure S3. PCA score plots of serum samples and QC samples in positive and negative ion modes. Figure S4. Z score plot of biomarkers identified in the model group and high-dose SQQX group. [file 8856943.f1.zip › Supplementary/Supplementary Figure.S2b. Overlapping TIC diagrams with QC samples-possitive mode.tif]
